# Immunological role of nasal *staphylococcus aureus* carriage in patients with persistent allergic rhinitis

**Published:** 2008-10-30

**Authors:** Maged Mohamed Refaat, Tarek Mansour Ahmed, Zeinab Ahmed Ashour, Mohamed Yousif Atia

**Affiliations:** 1 Internal Medicine department, Ain Shams University, Cairo, Egypt;; 2 Allergy and Immunology Research Unit Ain Shams University, Cairo, Egypt.

**Keywords:** nasal staphylococcus, allergic rhinitis, Egypt

## Abstract

Nasal carriage of staphylococcus aureus (S.aureus) exerts immunomodulatory effect in patients with atopic dermatitis and it may contribute to airway inflammation and allergic response in patients with allergic rhinitis. We Aim to investigate the frequency of nasal S.aureus carriage in patients with persistent allergic rhinitis and its possible influence on their symptoms and immune markers. We chosed 20 non smoker patients with house dust mite (HDM) allergy causing allergic rhinitis and 20 non smoker healthy subjects matched for age and sex. For all subjects rhinoscopy was done, skin prick test, nasal culture for S.aureus, nasal interleukin 4,nasal total IgE, serum total IgE and serum specific IgE(SSIgE) for HDM. Nasal S.aureus was detected in 16/20 patients (80%) and 5/20 (25%) in healthy subjects with highly significant statistical difference p<0.01. Correlation of nasal staph.aureus count and different systemic and local immune markers revealed highly significant positive correlation between nasal S.aureus count and serum total IgE (r = 0.78, p<0.01) and significant positive correlation with SSIgE (HDM) (r = 0.53, p<0.05), nasal total IgE (r = 0.39, p<0.05) and nasal IL-4 (r = 0.55, p<0.05). Nasal staph.aureus actively modulated the immune reaction in persistent allergic rhinitis patients by promoting local IgE production, so we recommend early detection and treatment of S.aureus carriage in patients.

Nasal carriage of staphylococcus aureus ( *S. aureus* ) exerts immunomodulatory effects in patients with atopic dermatitis and it may contribute to airway inflammation and allergic response in patients with allergic rhinitis. We investigated the frequency of nasal *S. aureus* carriage in patients with perennial allergic rhinitis and its possible influence on their symptoms and immune markers. Twenty patients with house dust mite (HDM) allergy causing allergic rhinitis and 20 healthy matched subjects underwent skin prick tests, symptom assessment, nasal culture for *S. aureus* , and measurement of nasal and serum immunological markers. Nasal *S.aureus* was detected in 16/20 patients (80%) and 5/20 (25%) in healthy subjects (p<0.01). We found positive correlation between nasal *S. aureus* counts and serum total IgE (r = 0.78, p<0.01), serum specific IgE (HDM) (r = 0.53, p<0.05), nasal total IgE (r = 0.39, p<0.05) and nasal IL-4 (r = 0.55, p<0.05) in allergic patients. Perennial allergic rhinitis sufferers have high rates of staphylococcal colonization putting them at risk of infection. Whether nasal carriage of *S. aureus* causes more severe allergy or vice versa remains to be shown.

The nose is regarded as the major site of Staphylococcus aureus *(S. aureus)* carriage from where the organisms spread. *S. aureus* grows within the nasal vestibule, close to the mucocutaneous junction. Nasal carriage of *S. aureus* plays a key role in staphylococcal infections [1] and may also constitute a risk factor for disease exacerbation in such rare conditions as Wegener's granulomatosis [2].

Staphylococcal colonization possibly modulates allergic disease. Patients with atopic dermatitis suffer from relapses of their disease following dermal overgrowth with this organism and most patients with atopic dermatitis develop IgE antibodies to staphylococcal antigens [3].

*S. aureus* can harbor superantigens (Sag), which exert immunomodulatory and proinflammatory effects [4] via activation of T-cells [5], B-cells [6], and mast cells [7] and enhance secretion of IL-4 and IL-5 [8] and production of IgE. Staphylococcal SAg have been shown to play a role in airway diseases [9, 9] and can contribute to airway inflammation and the development of airway hyper responsiveness in asthma [11]. Studies from Japan and Germany [12, 13] have shown higher carriage rates in patients with perennial allergic rhinitis (PAR). We set out to investigate the frequency of nasal *S. aureus* carriage in Egyptian patients with PAR and its possible influence on their symptoms and immune markers.

The cross sectional case control study was approved by the review board of the Allergy and Clinical Immunology Department, Ain Shams University, Cairo. We recruited 20 non-smokers with house dust mite (HDM) allergy causing PAR from the Allergy and Clinical Immunology outpatient clinic of Ain Shams University Hospital during the period of May to October 2006. Patients were 6 males (30%) and 14 females (70%) between 18 and 50 years of age. All had positive skin prick test to HDM. As controls we included 20 age and sex matched, healthy non smokers without past or family history of allergic disorders. All had negative skin tests for HDM and other common allergens. Exclusion criteria were pregnancy, lactation, ongoing or previous immunotherapy, asthma, emphysema, atopic dermatitis. Antihistamines and steroids were stopped three days before the study.

Nasal symptoms were scored according to the criteria of Okuda et al. [14] and Shiomori et al.[12]. A nasal lavage was performed after mucosal decongestion. Dilutions of this lavage were plated on blood agar and incubated at 36° Celsius in the presence of 5% C0 _2_ . *S.aureus* colonies were identified visually and confirmed by Gram stain and positive catalase reaction. Nasal IL-4, nasal and serum total IgE and specific HDM IgE were measured with commercially available immunoassays according to the manufacturers’ instructions.

The symptom scores of allergic patients were (mean (SD)): nasal blockage 2 (0.66), sneezing 2 (0.76), rhinorrhea 2 (1.2). Levels of immunological markers in patients and controls are shown in Table 1 . Nasal colonization with *S. aureus* was detected in16 of 20 (80%) patients and 5 of 20(25%) of controls (Chi square p<0.01). Bacterial counts were significantly higher in colonized patients than in colonized controls (mean 967.5 vs. 125, p<0.01).

We found positive correlation between nasal *S. aureus* counts and sneezing (r=0.44, p<0.05) but not with regard to other symptoms. In controls there was no correlation between staphylococcal counts and immunological markers. In contrast, in allergic patients, there was significant correlation between bacterial counts and serum total IgE (r=0.78, p<0.01), serum specific IgE (r=0.53, p<0.05), nasal total IgE (r=0.39, p<0.05) and nasal IL-4 (r=0.55, p<0.05) ( Figure 1 ).

Our colonization results were in line with previous studies by Shiomori et al.[12] and Riechelmann et al.[13], in that carriage rates are significantly higher in patients suffering from PAR than in healthy controls. This appears to be a global phenomenon. Also, like the former, we found correlation between bacterial counts and nasal symptoms. Such associations can apparently not be found in other types of allergic rhinitis [15].

We also found positive correlation between the number of colonizing staphylococci and several immunological markers in the nose and blood.

Several studies suggest that patients suffering from allergic rhinitis may have more difficulty fighting colonization. Zhou et al. [16] demonstrated that the immune adhesive function of leukocytes is decreased in PAR. Cole et al.[15] showed that staphylococcal colonization of patients with acute rhinitis leads to a local inflammatory response including antimicrobial defenses, but this response fails to clear the bacteria. Moreover, some strains of S. aureus carry superantigens which have been shown to inhibit the activity of T-regulatory cells that normally control inflammation [17]. In all, staphylococci might prefer to colonize very allergic noses, from where it is easy to spread via sneezing.

There is no doubt that perennial allergic rhinitis leads to more staphylococcal colonization and that this could put these patients at risk of more severe staphylococcal infections. It remains to be shown, however, if and how the presence of *S. aureus* actually modulates the severity of the rhinitis. We have to keep in mind that being seen at the scene of a crime does not mean that one has committed the crime. Further studies, such as attempts at eradication of staphylococcal carriage and reevaluation of symptoms and immunological markers are needed.

## Figures and Tables

**Figure 1:**
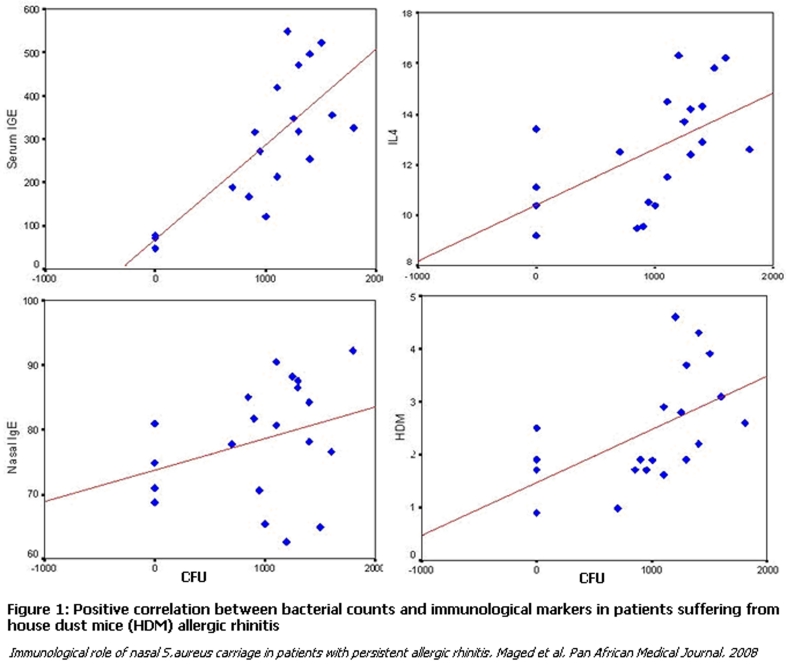
**
Positive correlation between bacterial counts and immunological markers in patients suffering from house dust mice (HDM) allergic rhinitis
** *
Immunological role of nasal S. aureus carriage in patients with persistent allergic rhinitis, Maged et al, Pan African Medical Journal, 2008
*

**Table 1:** Comparison of immunological markers between cases and controls

	Patients mean (SD)	Controls mean (SD)	p (t-test)

Serum total IgE (IU/ml)	280.6 (158)	13.8 (7.6)	<0.01
Serum HDM specific IgE (KU/ml)	2.44 (1.05)	0.17 (0.06)	<0.01
Nasal total IgE	78.4 (8.9)	32 (8.7)	<0.01
Nasal IL-4	12.6 (2.25)	5 (1.6)	<0.01

IU/ml: International Unit per milliliter, KU/ml: Killing units per milliliter, HDM: House Dust mite

*Immunological role of nasal staphylococcus aureus carriage in patients with persistent allergic rhinitis, Pan African Medical Journal, Maged et al. 2008*
